# Renal sympathetic denervation using MR-guided high-intensity focused ultrasound in a porcine model

**DOI:** 10.1186/s40349-016-0048-9

**Published:** 2016-02-03

**Authors:** Matthias Koopmann, Jill Shea, Eugene Kholmovski, Joshua de Bever, Emilee Minalga, Matthew Holbrook, Robb Merrill, J. Rock Hadley, Theophilus Owan, Mohamed E. Salama, Nassir F. Marrouche, Allison Payne

**Affiliations:** CARMA Center, Department of Cardiology, University of Utah, 30 North 1900 East, Salt Lake City, UT 84132 USA; Department of Surgery, University of Utah, 30 North 1900 East, Salt Lake City, UT 84132 USA; Department of Radiology, University of Utah, 729 Arapeen Drive, Salt Lake City, UT 84108 USA; Department of Bioengineering, University of Utah, 36 S. Wasatch Drive, Rm. 3100, Salt Lake City, UT 84112 USA; Department of Cardiology, University of Utah, 30 North 1900 East, Salt Lake City, UT 84132 USA; Department of Pathology, University of Utah, 15 North Medical Drive East Ste #1100, Salt Lake City, UT 84112 USA

**Keywords:** High-intensity focused ultrasound, Renal sympathetic denervation, MRI

## Abstract

**Background:**

Initial catheter-based renal sympathetic denervation (RSD) studies demonstrated promising results in showing a significant reduction of blood pressure, while recent data were less successful. As an alternative approach, the objective of this study was to evaluate the feasibility of using magnetic resonance-guided high-intensity focused ultrasound (MRgHIFU) to perform RSD in a porcine model.

**Methods:**

An intravascular fiber optic temperature probe was used to confirm energy delivery during MRgHIFU. This technique was evaluated both in a vascular phantom and in a normotensive pig model. Five animals underwent unilateral RSD using MRgHIFU, and both safety and efficacy were assessed. MRI was used to evaluate the acoustic window, target sonications, monitor the near-field treatment region using MR thermometry imaging, and assess the status of tissues post-procedure. An intravascular fiber optic temperature probe verified energy delivery. Animals were sacrificed 6 to 9 days post-treatment, and pathological analysis was performed. The norepinephrine present in the kidney medulla was assessed post-mortem.

**Results:**

All animals tolerated the procedure well with no observed complications. The fiber optic temperature probe placed in the target renal artery confirmed energy delivery during MRgHIFU, measuring larger temperature rises when the MRgHIFU beam location was focused closer to the tip of the probe. Following ablation, a significant reduction (*p* = 0.04) of cross-sectional area of nerve bundles between the treated and untreated renal arteries was observed in all of the animals with treated nerves presenting increased cellular infiltrate and fibrosis. A reduction of norepinephrine (*p* = 0.14) in the kidney medulla tissue was also observed. There was no indication of tissue damage in arterial walls.

**Conclusions:**

Performing renal denervation non-invasively with MRgHIFU was shown to be both safe and effective as determined by norepinephrine levels in a porcine model. This approach may be a promising alternative to catheter-based strategies.

## Background

Arterial hypertension represents a critical health challenge for millions of people, producing a well-established increased risk for an array of cardiovascular diseases affecting 74.5 million adults in the USA [1]. Appropriate adjustment of blood pressure is frequently challenging, despite the numerous pharmacologic options available. Indeed, roughly 40 % of patients undergoing treatment have uncontrolled hypertension [2]. A portion of this population has treatment resistant hypertension (TRH), which is identified in a patient when a therapeutic strategy of a diuretic and two other antihypertensive drugs fail to lower blood pressure values below 140/90 mmHg. While the prevalence of TRH in the uncontrolled hypertension population varies significantly in the literature, there appears to be an approximate prevalence of 10–20 % [3, 4]. Recognition of this common clinical problem has stimulated research exploring adjunctive non-pharmacological approaches. The well-characterized role of the sympathetic renal nervous system in initiating and maintaining hypertension [5] has led to the development of technologies that target and interrupt sympathetic renal nerves residing in the arterial wall and perivascular soft tissue.

Numerous pre-clinical and clinical trials have investigated endovascular catheter-based technologies as a primary or adjuvant treatment for TRH. Initial clinical studies reported promising results by significantly lowering both systolic and diastolic blood pressure [6, 7], even after 3 years of follow-up [8]. Those studies resulted in an increased interest in the technique and usage at multiple worldwide sites. However, a randomized, multicenter clinical trial applying catheter-based renal sympathetic denervation (RSD) in humans did not show a significant decrease in blood pressure when compared to the sham-control group [9]. Conversely, a prospective, open-label randomized control trial [10] demonstrated that in subjects treated with RSD in addition to a standardized stepped-care antihypertensive treatment (SSHAT) had reduced ambulatory blood pressure more than SSHAT alone.

Even though the catheter-based technologies have shown variable results, the procedure has demonstrated significant promise justifying the investigation of both catheter-based and other RSD treatment options.

High-intensity focused ultrasound (HIFU) is an established treatment option in various disorders [11] and has been proposed as an alternative energy delivery source for RSD therapy. Recently both an ultrasound and MRI-guided approach demonstrated feasibility using HIFU to perform RSD in normotensive canine [12] and porcine models [13] with mixed efficacy results. This study furthers those feasibility assessments by performing renal denervation using magnetic resonance-guided high-intensity focused ultrasound (MRgHIFU) in a normotensive porcine model.

## Methods

In MRgHIFU therapy, MRI is used in all aspects of the treatment process including planning, real-time procedure monitoring, and assessment [11]. Ideally, real-time MR thermometry [14] is used to measure the temperature elevation during the procedure and predict the tissue damage based on the accumulated thermal dose [15]. However, imaging artifacts due to the presence of motion (including arterial, respiratory, and peristalsis motion) and the presence of fat render standard proton resonance frequency thermometry techniques inaccurate [14] for monitoring temperature in the predominantly fatty tissue around the renal arteries. Because of these effects, obtaining accurate MR thermometry measurements in the area immediately surrounding the renal artery (i.e., regions extending approximately 1 cm away radially from the artery centerline) is extremely challenging. In this work, real-time MR thermometry measurements were not obtained in the regions immediately surrounding the renal artery during the RSD procedure. Therefore, in order to obtain a real-time assessment of the energy delivery to the target area surrounding the renal artery by the HIFU beam, an intravascular fiber optic temperature probe was placed in the targeted artery and continuously monitored during the RSD procedure. The use of this invasive temperature probe was evaluated in a vascular phantom as well as an in vivo normotensive porcine model.

### Vascular phantom preparation

**Fig. 1:**
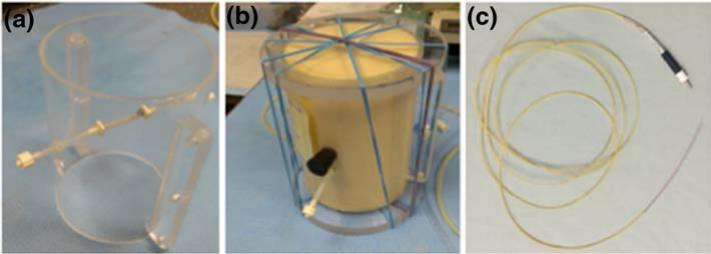
Vascular phantom construction. **a** Picture of vascular phantom mold with excised rabbit aorta and fiber optic temperature probe in place. **b** Same vascular phantom after gelatin was poured around the vessel. **c** Fiber optic temperature probe used in both the phantom and in vivo pig experiments

In order to validate the use of an intravascular temperature probe, a vascular phantom was developed. Figure 1a shows an excised rabbit aorta secured in an acrylic phantom mold. A fiber optic temperature probe (Neoptix, Quebec, Canada) was placed in the vessel such that fluid could flow around the probe through the vessel, and tissue-mimicking gelatin was poured around the vessel [16]. The phantom was mounted on a pre-clinical MRgHIFU system (256-element phased-array transducer, *f* = 1 MHz, 2 × 2 × 8 mm focal spot size, Image Guided Therapy, Inc., Pessac, France), and the entire assembly was placed in a Siemens Trio 3 Tesla MRI scanner (Erlangen, Germany). Degassed, deionized water was used to both acoustically couple the phantom to the transducer and to perfuse the embedded vessel.

**Fig. 2:**
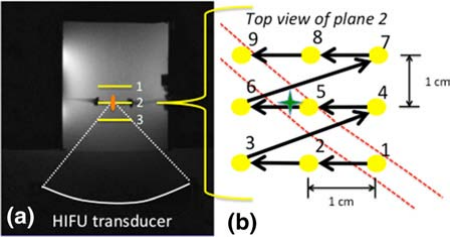
Sonication pattern in the vascular phantom. **a** Axial MR image of gelatin vascular phantom placed over focused ultrasound transducer. Three planes of a nine-point raster pattern were sonicated centered on the vessel. **b** Top view of a single nine-point raster pattern. The approximate location of the vessel is shown by the *dashed lines*. The approximate location of tip of the fiber optic probe is indicated by the *green star*. Spacing between the points in plane and between planes was 1 cm

Multiple sonications were performed in a three-plane, 27-point raster pattern with 1 cm spacing centered on the embedded excised artery at two flow rates that allowed for flow past the intravascular probe, 40 and 80 mL/min (Fig. 2). Each point was sonicated for 20 s at 35 W and 20 s of cooling time elapsed before the following point was sonicated. The fiber optic temperature probe recorded the temperature in the artery every 0.5 s. MR thermometry during the experiment was achieved with a 3D segmented-EPI gradient echo sequence (TR/TE = 40/10 ms, flip angle = 40°, 1.6 × 1.6 × 3 mm resolution, 112 × 256 × 24 mm FOV, ETL = 9. Two separate 2-channel surface RF coils were placed on the sides of the cylindrical phantom holder to provide sufficient SNR for the study.

The position of each focal spot was determined by the location of the peak temperature as measured by the MR temperature imaging (MRTI). The temperature rise (*T*_rise_ = *T*_*peak*_ − *T*_baseline_) detected by the fiber optic probe at each sonication location was also determined.

### Animal preparation

All applicable institutional and national guidelines for the care and use of animals were followed. Five normotensive female Yorkshire pigs (40–50 kg) were included in the study. Anesthesia was induced with a Telazol, Ketamine, and Xylazine cocktail (4.4, 2.2, and 2.2 mg/kg, respectively) and maintained with isoflurane (1–3 %, inhaled). Hair on the back of the animal was removed with clippers and a depilatory cream to improve acoustic window quality.

Similar to the vascular phantom, a fiber optic temperature probe was placed in the right renal artery through percutaneous access of the femoral artery under fluoroscopy guidance. The temperature probe was sheathed in a 6-French multipurpose angiographic catheter with the tip of the temperature probe extended approximately 1 cm distal to the end of the angiographic catheter.

### MRgHIFU renal sympathetic denervation procedure

**Fig. 3:**
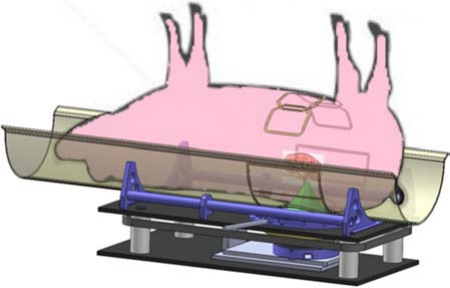
Schematic of pig placement on MRgHIFU device. The position of the transducer below the animal with the cone depicting the ultrasound focus. The positioning of the nine RF receiver coils is also shown

RSD in the porcine model was performed using the same pre-clinical MRgHIFU system and MRI scanner as in the vascular phantom study. The animal was placed on top of the MRgHIFU system in a custom support holder in an oblique supine position with an integrated 9-channel RF receive coil surrounding the animal (seen schematically in Fig. 3). MR imaging was used to accurately position the animal, evaluate the acoustic window, and plan the sonication locations around the target renal artery (3D T1-weighted Volumetric Interpolated Breath-hold Examination [VIBE], T2-weighted Turbo Spin Echo [TSE]).

Because of the location of the bowel in all the animals treated in this study, RSD using MRgHIFU was performed in all animals unilaterally on the right side, with the left side serving as a control. Several single-point sonications (as detailed in Table 1) were applied to the regions at a close anatomical proximity to the right renal artery. In general, the number of sonications applied per animal was a function of the overall length of the renal artery and the available study time. While the transducer power output was approximately 80 W for animals 1 through 3, the power was increased in animals 4 and 5 to 110 and 140 W, respectively. The animal’s SpO_2_, end tidal CO_2_, and body temperature were monitored continuously throughout the MRgHIFU procedure.

**Table 1:** MRgHIFU sonication details for each of the treated animals

Animal ID	No. of sonication points	Sonication time/point (s)	Acoustic power (W)	Total energy (kJ)	Δtime (days)
1	7	20	83	11.6	6
2	26	20	81	42.1	6
3	17	20	82	27.9	7
4	16	20	120	38.4	9
5	16	45	140	100.8	9

Δtime indicates time between the RSD procedure and necropsy

**Fig. 4:**
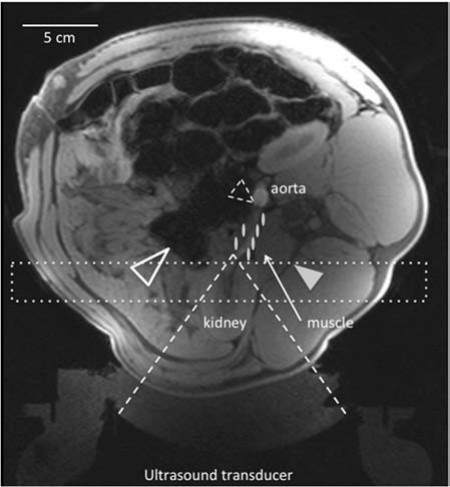
Axial T1-weighted MR image of animal 3. The acoustic window of the ultrasound transducer targeting the right side of the animal is shown by the *dashed lines*. The approximate locations of six of the 17 sonications are shown as *white ovals* surrounding the right renal artery. The MR images were used to target the tissue surrounding the artery avoiding the transverse process (*solid arrow*) and bowel (*hollow arrow*). The angiographic catheter can be seen in the aorta and renal artery (*hollow dashed-arrow*). The *dotted box* shows the approximate location of the field of view monitored during the RSD procedure

Due to the significant susceptibility artifacts from peristalsis, blood flow artifacts, and the presence of fat in the target region, temperature measurements in the area immediately surrounding the renal artery were not obtained in this study. However, MR thermometry techniques were used to monitor the treatment in the near field of the ultrasound beam. The 3D imaging volume, as indicated in Fig. 4, was placed such that any interference between the ultrasound beam and transverse process could be monitored using real-time MRTI (3D Segmented-Echo Planar Imaging [EPI]). The MRTI measurements were used to calculate the thermal dose, as defined by Sapareto and Dewey [15], deposited in the tissues during the course of the MRgHIFU RSD treatment. T2-weighted TSE and post-contrast VIBE scans (0.05 mmol/kg, MultiHance, Bracco Diagnostics Inc.) were used to evaluate surrounding tissues post-procedure. Relevant MR parameters for all listed sequences are located in Table 2.

**Table 2:** Typical MRI parameters used in the in vivo experiments

Pulse sequence	TR (ms)	TE (ms)	Flip angle (°)	Resolution (mm)	FOV (mm)
3D T1w VIBE	4.33	1.97	9	1.2 × 1.7 × 3	380 × 286 × 168
2D T2w TSE	2000	89	180	1.3 × 1.4 × 4	320 × 280 × 72
3D seg-EPI MRTI	35	11	25	2 × 2 × 3	256 × 192 × 30

### Tissue processing

Six to 9 days after the renal denervation procedure, the animal was sacrificed and a necropsy performed. Bilateral kidneys, renal arteries and surrounding tissue, abdominal aorta, and adjacent muscle were examined for any gross abnormalities. Tissue was fixed for 24 to 48 hours in 10 % formalin. Each renal artery was divided into four equal segments with the segment closest to the aorta designated as region 1 and the segment closest to the kidney designated as region 4. The segments were dehydrated in increasing concentrations of alcohol, embedded in paraffin, and then sectioned (5 μm). One hematoxylin and eosin (H&E) slide per segment was prepared and analyzed.

### Morphometric analysis

The stained sections were digitally scanned with the ScanScope® XT system and visualized using ImageScope software in eSlideManager (Aperio/Leica Biosystems, Vista, CA) [17, 18]. Each arterial segment (regions 1–4) was analyzed using positive pixel count and measurement tools of ImageScope software to determine nerve count, cross-sectional nerve and artery area, and distance from nerve to arterial lumen. For calculation and analysis of mean nerve area, only nerves that were greater than 5000 μm^2^ and smaller than 70,000 μm^2^ were included in the calculation.

### Norepinephrine-ELISA

At necropsy, both kidneys were immediately placed in an ice-cold phosphate buffered saline, and segments of the medulla were isolated, weighed, homogenized in 0.8 M EDTA, and then frozen (−80 °C). The levels of norepinephrine (ng/mL) in the homogenate were measured via enzyme-linked immunosorbent assay (ELISA) following the manufacturer’s instructions (Rocky Mountain Diagnostics, Colorado Springs, CO).

### Statistics

Nerve area and kidney norepinephrine (NE) levels were compared between the treated and non-treated sides with a paired *t* test (JMP Pro 11; SAS; Cary, NC), with significance set at *p* < 0.05.

## Results

### Vascular phantom

**Fig. 5:**
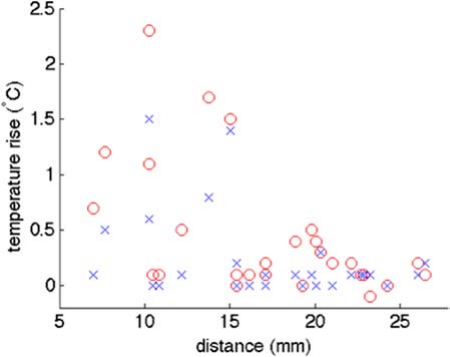
Vascular phantom thermal response. Peak fiber optic temperature change measured in the vascular gelatin phantom during each sonication as a function of distance between the focused ultrasound beam location and fiber optic probe tip. The two tested flow rates, 80 mL/min (*blue x*) and 40 mL/min (*red o*) are shown

The results shown in Fig. 5 from the vascular phantom experiments demonstrate that MRgHIFU sonications performed closer to the tip of the fiber optic temperature probe resulted in a higher measured temperature rise. This decreasing trend of temperature rise as a function of sonication distance from the probe tip to the focused ultrasound beam location is seen at both the 40 and 80 mL/min flow rate. Predictably, overall higher temperature rises were observed at the lower flow rate.

### MRgHIFU RSD procedure

A representative pre-RSD treatment acoustic window evaluation using T1-weighted (T1w) 3D VIBE images, which is utilized to evaluate effective transducer positioning and acoustic coupling of the transducer to the animal’s skin, is shown in Fig. 4. The spine, bowel, kidney, aorta, and renal artery are all easily visualized without contrast agent allowing the animal to be positioned such that the interaction of the ultrasound beam with high acoustic impedance anatomy was minimized. The angiographic catheter housing the fiber optic temperature probe is seen in the aorta and at the renal artery junction.

**Fig. 6:**
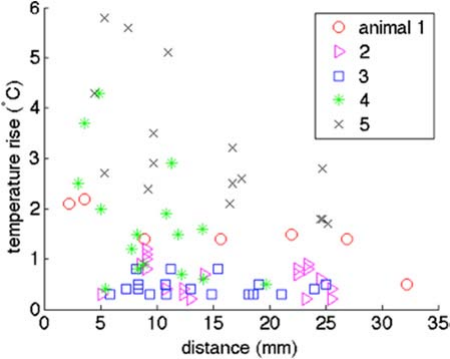
Porcine model thermal response. Peak fiber optic temperature change measured during the RSD procedure in each of the five animals. Decreasing trends of temperature rise as a function of distance from the fiber optic probe tip to the focal spot position was observed in all animals

The fiber optic temperature probe placed in the renal artery on the treated side provided verification of energy delivery that was independent of MR measurements. The temperature rise measured by the probe as a function of distance to the targeted MRgHIFU beam location is shown in Fig. 6. Similar to the observations made in the vascular phantom, the temperature rise measured by the fiber optic temperature probe decreases as the distance between the probe tip and the MRgHIFU beam location increases. While the magnitude of this relationship varies, as seen in Table 3, the trend is present for all evaluated animals. In addition, the magnitude of the temperature rise increases with increased power output from the transducer.

**Table 3:** Procedure results for all treated animals

Animal ID	Fiber optic temperature probe		Near-field MRI measurements	
	Slope (°C/mm)	*R* ^2^ value	Edema (y/n), volume (mm^3^)	Volume (mm^3^) ≥240 CEM 43 °C
1	−0.04	0.74	No	125
2	−0.007	0.020	Yes, 269	607
3	−0.004	0.016	No	25
4	−0.13	0.27	Yes, 774	1002
5	−0.12	0.47	No	123

*Slope* is the decreasing temperature trend as a function of distance from fiber optic probe tip to MRgHIFU focus location

**Fig. 7:**
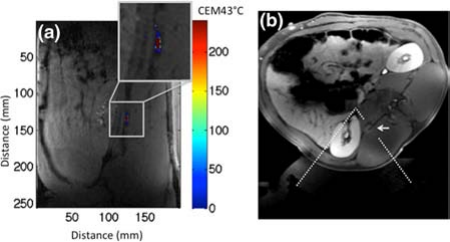
MRI monitoring of the renal denervation procedure. **a** Coronal view of a plane in the near field of the ultrasound beam for animal 7. The *enlarged inset* indicates an area that accumulated thermal dose with potential necrotic damage. The total volume with potential damage in this animal was 123 mm^3^. The values for all animals are given in Table 3. **b** Corresponding enhancement around the transverse process denoted by the *white arrow* is seen at the slice location in a post-ablation delayed contrast-enhanced T1w image. The approximate insonified area is represented by the *dashed white line* (a gap is present so as to not obscure the non-enhancing area)

The real-time MRTI monitoring that was performed in the near field of the MRgHIFU beam confirms that in all animals, some energy was deposited in the muscle area surrounding the transverse process. Figure 7a shows the cumulative thermal dose deposited during an RSD procedure overlaid on a coronal magnitude image. The volume of tissue in the near field that received possible permanent damage (thermal dose > 240 CEM43°C [19]) ranged from 25 to 1000 mm^3^ as listed in Table 3. This potential damage was confirmed by delayed contrast-enhanced T1w VIBE image (Fig. 7b). In two out of five of the animals, the presence of edema was detected by post-RSD T2-weighted imaging. The existence of edema and the corresponding size of the enhancing regions are reported in Table 3.

### MRgHIFU RSD procedure safety

All animals recovered quickly from the RSD procedure with no observed complications. During necropsy, all anatomical structures between the energy source and the target region were carefully observed including the skin, muscle tissue, spine, renal arteries and veins, ureters, liver, bowels, and kidneys. Based on gross histological examination, there was no detectable tissue damage along the acoustic beam, other than in the target region. Importantly, injuries of the arterial wall were not observed.

Gross examination revealed several hemorrhagic spots located in the fatty tissue around the treated renal arteries. The length of the renal artery from the aorta to the bifurcation was not found to be significantly different (*p* = 0.17) between the treated (3.4 ± 0.5 cm) and the control side (3.1 ± 0.2 cm). The distance from the nerves to the lumen (endothelium) of the renal artery was determined for both the treated and control sides (Table 4). A total of 83 nerves on the treated side and 69 nerves on the control side (Table 4) met the inclusion criterion. Thirty-nine nerves that were smaller than 5 μm^2^ on the treated side and 49 on the control side were excluded. There were 14 nerves on the treated side that exceeded 70 μm^2^ and 12 on the control side. The majority of the nerves were located within 3 mm from the lumen of the artery (90 % control and 96 % treated). Regionally, a majority of nerves were located in regions 3 and 4, closer to the renal pelvis, both on the control (73 %) and treated (71 %) sides. There was also no significant difference in renal artery area between the treated side (6.03 ± 1.53 mm^2^) and the control side (6.70 ± 2.04 mm^2^, *p* = 0.27). There were no histological indications of damage to the renal artery as a result of the MRgHIFU RSD procedure.

**Table 4:** Distance from the renal nerves to the endothelium of the renal artery as a function of anatomical position for treated and untreated arteries

Distance from lumen (mm)	Treated arteries				Control arteries			
	Region 1	Region 2	Region 3	Region 4	Region 1	Region 2	Region 3	Region 4
0–1	1 (1.2 %)	–	–	1 (1.2 %)	–	–	3 (4.4 %)	1 (1.5 %)
1–2	5 (6.0 %)	10 (12.1 %)	20 (24.1 %)	28 (33.7 %)	2 (2.9 %)	11 (15.6 %)	20 (29.0 %)	14 (22.3 %)
2–3	2 (2.4 %)	–	6 (7.2 %)	2 (2.4 %)	2 (2.9 %)	5 (7.3 %)	2 (3.4 %)	6 (8.7 %)
3–4	1 (1.2 %)	–	4 (4.8 %)	–	–	–	–	3 (4.4 %)
>4	–	3 (3.6 %)	–	–	–	–	–	–
Nerves/region	9	13	30	31	4	16	25	24

Each table cell represents the number of nerves visible in a single slide prepared from the designated region with the percentage of nerves for that given side. There is a proximal to distal distribution, while region 1 is closest to the aorta and region 4 closest to the kidney

### MRgHIFU RSD procedure efficacy

**Fig. 8:**
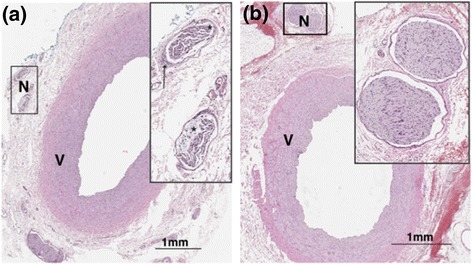
H&E stained sections of the **a** treated and **b** control arteries in animal 7. *Inset* (*N*) indicates the arterial nerves. Nerves damage is present in the treated side as exhibited by perineural fibrosis (*arrow*) and degradation of the nerve fibers (*asterisk*). There was no apparent damage to either of the vessels (*V*)

Cumulative nerve area on the treated side was statistically smaller than the cumulative nerve area on the control side, with all of the animals treated with MRgHIFU having reduced nerve area on the treated side (Table 5, *p* = 0.04). The mean nerve area on the treated side was roughly 25 % smaller than the control side (Nerve Area_treated_/Nerve Area_control_ = 0.74 ± 0.14, Table 5). Figure 8 shows the morphological changes observed, with the nerves on the treated side having increased cellular infiltrate, fibrosis, and shrunken appearance, all of which indicate damage to the nerve. The ratio of norepinephrine in the treated and control kidneys decreased in all five of the animals evaluated (Table 5), though this decrease was not found to be statistically significant between the treated and non-treated side (*p* = 0.14). The absolute values for norepinephrine ranged from approximately 500–1800 on the treated side and 1000–3300 on the control side as shown in Table 5.

**Table 5:** Ratio of treated to control arteries for different outcome measures

Animal #	1	2	3	4	5
Energy delivered (kJ)	21.6	42.1	27.9	38.4	100.8
Nerve area ratio	0.76	0.83	0.50	0.81	0.80
Medulla norepinephrine, treated side (ng/ml/g)	1147	1573	1813	525	1155
Medulla norepinephrine, control side (ng/ml/g)	1269	1827	2192	1060	3342
Medulla norepinephrine ratio	0.9	0.86	0.83	0.50	0.35

## Discussion

### MRgHIFU RSD efficacy

This study has demonstrated the feasibility of using MRgHIFU to perform RSD in a normotensive porcine model safely, resulting in nerve bundle damage. The norepinephrine measured directly from the kidney medulla tissue was reduced post-RSD procedure when comparing the treated kidney with the contralateral control kidney indicating successful RSD was performed [20]. While the number of animals treated in this feasibility study was small, the norepinephrine ratio generally decreased as the applied energy increased indicating a potential dose effect that should be explored further in future studies. This preliminary finding agrees with RSD procedures performed with catheter methods. In the Symplicity HTN-3 trial [9], there was a positive correlation between the number of ablation attempts and the decrease of blood pressure. The reduction seen in the norepinephrine data is supported by the histological appearance of damaged renal nerves. In addition, the cross-sectional area of the nerve was reduced by approximately 25 % on the treated side. This result is similar to other studies [21, 12] that have shown that nerve atrophy is a common indication of nerve damage, as observed following renal ablation and other common nerve injures and nerve injury models [22].

While the difficulties of obtaining accurate MR thermometry data at the treatment area prevented acute assessment of the success of the MRgHIFU procedure, the independent temperature measurements assessed with the intravascular fiber optic temperature probe provided confirmation of energy delivery. While the temperature rise measured by the probe for each sonication point did exhibit both inter- and intra-animal variability, in general, higher temperature rises were measured when the MRgHIFU beam focus was located close to the probe tip. Obviously, one of the main advantages of performing RSD with MRgHIFU is that the procedure could be completely non-invasive. Therefore, while using an intravascular fiber optic probe when performing RSD with MRgHIFU would not be ideal in future clinical work, this study has demonstrated that it can provide valuable information and qualitative treatment confirmation in pre-clinical studies. Therefore, while MR thermometry was not able to predict an acute treatment assessment, the use of the temperature probe did demonstrate the MRgHIFU beam was focused in close proximity to the renal artery. This result extends the assessment that has been performed in other HIFU RSD studies [13, 12].

This study did not compare blood pressure measurements before and after the RSD procedure. Similar to other work, we found separating the effect of the RSD procedure and anesthesia on blood pressure [23] to be quite difficult. Indeed, whether RSD affects blood pressure in normotensive animals remains a matter of debate [12]. For these reasons, kidney medulla norepinephrine concentration is reported as the primary efficacy outcome for this study, a proven robust marker for effective renal nerve destruction [20]. The norepinephrine reduction ranging from 10 to 65 % post-RSD MRgHIFU procedure compares to other clinical studies [6] where analysis from 10 patients revealed a mean reduction in norepinephrine spillover of 47 % at 1 month after bilateral RSD. These numbers also compare to other pre-clinical RSD studies performed with HIFU studies. In Wang et al. [12], a 51 % reduction in plasma norepinephrine was observed 6 days post-procedure. Conversely, in Freyhardt et al. [13], no significant change was observed in the renal parenchyma norepinephrine concentration.

### MRgHIFU RSD safety

While edema around the transverse process was observed in two animals with the largest thermal dose accumulations, no tissue effect was observed during necropsy. Although the majority of the entire kidney is in the near field of the ultrasound beam, as seen in Fig. 5, there was no observable damage to the organ. In addition, since the focal spot of the transducer is ellipsoid shaped approximately 2 × 2 × 8 mm in size, it is likely that the MRgHIFU beam focus may have directly targeted the renal artery. Despite this possibility, there was no indication of renal artery wall damage in any of the analyzed histological sections.

The real-time monitoring of the near-field regions during the MRgHIFU RSD treatment may potentially increase the safety of the overall procedure. Other studies have documented the potential of near-field heating buildup [24], particularly in cases where multiple sonications are executed from a fixed acoustic window, as was the case in this study.

### Model applicability

A porcine model was selected for this study due to similarities of the porcine cardiovascular system to human anatomy [25]. In this study, the highest nerve bundle density is at the distal part of the renal artery, close to the kidney hilum. However, others have also reported the opposite with more nerve fibers closer to the aorta [26, 27]. This variability of results indicates that when conducting an ablation procedure, it will likely be more effective if a greater region of the nerves around the artery is ablated to account for inter-patient variability.

Other anatomical features including the bowel and spinal column vary quite substantially between humans and porcine. The vertebrae of the porcine spinal column exhibits prominent transverse process potentially causing some local absorption of the acoustic beam. This effect was observed in two of the animals as assessed by the presence of edema post-RSD procedure. Conversely, in humans, the distance of the bowel to the left renal artery is not as close as in pigs. This difference would allow for bilateral renal artery ablation in humans. Indeed, human trials with ultrasound-guided HIFU are ongoing (clinicaltrials.gov, NCT02029885).

While the goal of RSD is to destroy the renal artery nerves with a negligible amount of collateral damage, it is difficult to determine the damage mechanism in this study. In our study, the total delivered energy per animal varied from 10–100 kJ. Other RSD HIFU studies reported total energy delivery of 18 kJ [12] and a mean of 26.2 kJ per animal [13] with varied efficacy results. This variability indicates that successful treatment outcome is a function of applied dose as well as animal position and size.

### Study limitations

Normotensive animals were used in this study and were treated unilaterally, which likely limits the efficacy results observed. Due to the location of the bowel, only the right side could be treated introducing a potential bias in the study. No conclusions can be made regarding the long-term effects of RSD performed with MRgHIFU since the longest time span from ablation to renal nerve and kidney tissue analysis was 9 days. We are currently exploring this question in ongoing pre-clinical studies. In addition, it should be noted when norepinephrine levels are assessed directly from the kidney tissues as done in this study, it does not allow the comparison of norepinephrine levels pre-RSD MRgHIFU procedure. There is the possibility that the reduction of norepinephrine may be due to other physiological changes including a change in stress level or vasoconstriction. However, in spite of these potentially confounding factors, the encouraging reduction in norepinephrine in the kidney medulla between the treated and control sides indicated that there was a dose ranging effect, which provides useful information to guide future study design.

### Potential advantages of MRgHIFU

MRgHIFU is a completely non-invasive technology that has the potential of being a valid RSD procedure technique. While arterial damage [28, 29] during catheter-based techniques has been rare, MRgHIFU would have no impact on vascular structure. It would also overcome any issues with renal artery anatomy [4]. In addition, performing the procedure under MR guidance can allow for detailed treatment planning and monitoring as well as a non-contrast angiographic method [30].

## Conclusions

This study demonstrates the feasibility of performing RSD using MRgHIFU in a porcine model. Soft-tissue contrast achieved by MR guidance is advantageous in pre-procedural planning, ensures accurate targeting, and allows for detailed visualization of the region of interest. While MR thermometry provided real-time monitoring of critical adjacent structures in the near field during the procedure, an intravascular fiber optic temperature probe provided real-time feedback at the target area. MRgHIFU has the potential to be a valid technique for non-invasively performing RSD. Future studies will evaluate this approach in a hypertensive animal model with a longer follow-up, and efforts will be made to improve MR thermometry techniques around the renal arteries.
